# Shared decision-making for people living with dementia in extended care settings: protocol for a systematic review

**DOI:** 10.1136/bmjopen-2016-012955

**Published:** 2016-11-02

**Authors:** Rachel Daly, Frances Bunn, Claire Goodman

**Affiliations:** Centre for Research in Primary and Community Care (CRIPACC), University of Hertfordshire, Hatfield, UK

**Keywords:** PRIMARY CARE

## Abstract

**Introduction:**

Approximately 450 000 people in the UK are living in care homes, 70% of whom are thought to have dementia or significant memory problems. This means that they may need support with day-to-day decisions about their health and care. Shared decision-making interventions can have a positive impact on patient outcomes. They recognise an individual's rights to make decisions about their care or treatment and support person-centred approaches to care delivery.

**Methods:**

A systematic review of studies designed to assess, implement, measure and/or explore shared decision-making with cognitively impaired adults in (or transferrable to) an extended care setting, with a view to answering the research question: How can people living with dementia and cognitive impairment be included in day-to-day decisions about their health and care in extended care settings? The systematic review will be started in May 2016. Studies are excluded that focus on advance decision-making. The search strategy is limited to a 20-year timeframe and English language and includes electronic databases; CINAHL, PubMed, the Cochrane Library, NICE Evidence, OpenGrey, Autism Data, Google Scholar, Scopus and MedicinesComplete.

**Ethics and dissemination:**

Ethical approval not required. Planned dissemination routes for protocol and systematic review through conference presentations, peer-reviewed journals and research networks including the East of England CLAHRC, INTERDEM, and the National Care Homes Research and Development Forum.

**Discussion:**

The review will explore how shared decision-making is characterised and constructed in extended care settings for people living with cognitive impairment and their staff and family carers, in relation to their preferences and desires, the roles people play, facilitators, barriers, risk and benefits. The findings will inform an intervention study facilitating shared decision-making for people living with dementia in care homes and have the potential to inform future policy and practice.

**Trial registration number:**

CRD42016035919.

**Strengths and limitations of this study**

- Provision of a contemporary synthesis of evidence relating to recognised risks and benefits of shared decision-making for people with a cognitive impairment and their carers in extended care environments.
- Creation of a robust interdisciplinary baseline outlining existing resources, tools and methods used to understand, facilitate and promote shared decision-making for people living with a cognitive impairment in extended care settings.
- Reporting bias at study level, for example, unsuccessful implementation studies are less likely to be published.
- Bias related to data extraction techniques, analysis or reporting methodologies at outcome level and potential inability to retrieve all relevant research due to the search strategy design.

## Background

Despite increasing international recognition of the need for shared decision-making in health and social care, and its potential impact on quality of life and the global health economy^1^ there is limited evidence of how it is used to support people living with dementia in care homes.^2^

This protocol defines each element in turn: dementia, extended care and shared decision-making before discussing the complex concept of shared decision-making for people living with dementia in extended care environments, and the factors that are known to influence it. Gaps in current knowledge will be identified along with how the review proposes to address those gaps.

### Dementia

Dementia describes a collection of symptoms that present when the brain is affected by disease processes that include, for example, Alzheimer's, Lewy body or vascular dementia. Symptoms are degenerative and individual but typically may include memory loss, personality changes and difficulties with word finding or problem-solving. For people over 55 years of age, dementia is more feared than any other health condition including cancer and diabetes.^3^ There are estimated to be 850 000 people living with dementia in the UK, rising to over 1 million in 2025 and 2 million by 2051.^4^

A psychosocial theory of dementia frames how the involvement of people living with dementia is viewed, recognising the importance of personhood, and that social and relational losses (not only progressive cognitive impairment) can diminish the personhood and self-worth of those living with dementia.^5^ ^6^

### Extended care settings

For the purposes of this review the term ‘extended care setting’ is used to include all types of residential housing with onsite care provision. In addition to care homes, extended care settings include supported living, care villages and extra care housing. Approximately 450 000 people live in care homes in the UK^7^ and Prince and colleagues estimate that around 70% of care home residents in the UK have dementia or significant memory problems; as a result this population may need support and assistance with decisions about their day-to-day health and care.

### Shared decision-making

Shared decision-making is a partnership which enables clinicians and patients to make health and care-related treatment, management or support decisions based on best available clinical evidence and the patient's own values and preferences. It involves eliciting the patient’s ideas, concerns and expectations^1^ and the provision of evidence-based information about options, outcomes and uncertainties.^8^

Decision support tools or aids clarify available treatment options, including possible harm and benefits, and support people to work with professionals to choose a course of care that reflects their personal values. Internationally a variety of tools have been developed to support shared decision-making^9–11^ especially in relation to specific healthcare screening and interventions.^12–14^ Shared decision-making has been recognised as having a positive impact on a range of patient outcomes.^8^ ^15^ ^16^

This review will explore the role of shared decision-making in day-to-day health and care decisions between (staff and family) carers and people living with dementia in extended care settings. For example, this might include decision-making about personal care preferences, medication regimes, or the timing and approach to changing a wound dressing. Some of these more seemingly trivial decisions need to be faced each day and for a person living with dementia who may be dependent on help and support from others to fulfil their care needs and desires, *how* decision-making is approached, understood, and negotiated that can be indicative of the impact on their personhood. The opportunities for choice and control and the perceived risks and benefits of any given decision may be largely dependent on the relationship between the person living with dementia and their carers.^1^ ^2^

Central to the topic of shared decision-making for a person living with dementia is their ability to make their own decision, either with or without support. Successful shared decision-making assumes that care receivers are informed, empowered and enabled to participate in discussions about their health and care. It requires them to have developed the skills, knowledge and confidence required to discuss their options with experts, challenge professional views, and influence their care and outcomes.^15^ This may prove a significant challenge for individuals living with dementia. The possibilities for, and appropriateness of, shared decision-making for people with cognitive impairments has been researched within the field of acute mental health and there is evidence of positive outcomes for all involved, including improved knowledge, well-being and medication adherence in addition to reduced conflict.^17–19^

The historical assumption that people living with a cognitive impairment cannot participate in decision-making is increasingly being challenged and an individual living with a cognitive impairment's ability to maintain active participation in decisions about their health and care has caused considerable debate.^20–22^ In practice, many settings rely on family members to make care decisions for people living with dementia, often regardless of individual's currently stated preferences, legal, medical or ethical processes.^16^ In their review of international literature on patient and carer involvement in shared decision-making for people living with dementia, Miller and colleagues acknowledged that research in this area of practice is relatively new; however, they identified multiple sources of evidence which indicates that people with dementia can reliably report on their ideals and preferences in relation to their care, well-being and quality of life, even through moderate-to-severe dementia. Therefore, while family carer involvement is essential, it should be sought as a partner and not to supersede the views of the person living with dementia. The review focused on shared decision-making within ‘family care dyads’ in the community (comprised of a person with dementia and a family carer) although care dyads might equally comprise a health or social care professional and a person living with a cognitive impairment. Regardless of the other parties involved, the person living with the cognitive impairment must, at least, be given an opportunity to choose to participate in the decision-making process.^16^ Furthermore, many people living with dementia maintain their ability to communicate their values and preferences albeit through verbal, non-verbal and tailored communication aids, long after their executive decision-making ability is affected by cognitive decline.^16^

Extensive work undertaken in the UK by the Dementia Action Alliance^23^ has identified that people living with dementia want personal choice and control in decisions that affect them, and to know that services are designed to meet the needs of themselves and their carers. Person-centred care is now widely accepted as the method for ensuring individuals are involved in planning and designing their own care and is an ethical and legal requirement throughout Europe, Australia and North America.^1^ ^24^ ^25^ It is also embedded in the UK national policy and health and care regulations (MCA, 2005; DH, 2010; Care Act, 2014) and international guidance.^26^ To abide by the law and fulfil the moral obligation to provide person-centred care, it is important to have an understanding of each person's needs and desires and, where possible, to include them in all the decisions that shape their care. This is reflected in the drive for improved treatment of people living with dementia and their carers^4^ and greater involvement in the decisions central to their care.^27^

## Methods

The review will be conducted using methods outlined in the Cochrane handbook of systematic reviews of interventions.^28^ This protocol has been designed in accord with the Preferred Reporting Items for Systematic Reviews and Meta-Analyses (PRISMA-P) guidelines^29^ and checklist (see online supplementary file 1). The protocol is registered with PROSPERO international prospective register of systematic reviews registration number CRD42016035919.

The aim of the review is to understand how day-to-day decisions are negotiated between people with a cognitive impairment and their (staff and family) carers in extended care settings, with a view to gaining transferrable learning that can be applied to people living with dementia in care homes.

The review objectives are to:

- Explore how shared decision-making is understood and/or characterised for people living with dementia and their (staff and family) carers;
- Explore the role of (staff and family) carers of people living with dementia in shared decision-making care dyads;
- Analyse identified risks and benefits associated with shared decision-making for people with cognitive impairment;
- Ascertain empirical evidence for the effectiveness of available shared decision-making resources for people living with dementia;
- Seek to understand the barriers and facilitators to effective shared decision-making for people living with dementia and their (staff and family) carers;
- Explore the extent to which shared decision-making has been researched in extended care settings;
- Identify implications for shared decision-making in dementia care practice, policy and future research.

### Inclusion criteria

#### Participants

The focus of the review is adults, over 18 years, living with any type of dementia in an extended care setting. Studies relating to adults with other cognitive impairment (eg, learning disability or brain injury) will be included where the model, tools or intervention is transferable to people living with dementia in an extended care setting. To be considered transferrable, the person living with a cognitive impairment must be in receipt of care in addition to their family carer and the intervention, measure, resource or method should be able to be practically implemented within an extended care setting. Authors will discuss and agree by consensus if there is any doubt regarding the inclusion of any paper.

#### Setting

The term ‘extended care setting’ is used to include all types of residential housing with onsite care provision. The UK Care Quality Commission define care homes as offering ‘accommodation and personal care for people who may not be able to live independently’ and register care homes ‘with’ and ‘without’ nursing.^30^ Studies in other settings, for example, people's own homes, will be included if they meet all other inclusion criteria and are transferable to an extended care setting.

#### Interventions

Studies will be included if they report primary research designed to assess, implement, measure and/or explore shared decision-making with cognitively impaired adults. With particular focus on interventions relating to day-to-day health and/or care decision-making (eg, decisions relating to personal care or medication management).

### Exclusion criteria

Papers specific to advance decisions or advance care planning will be excluded as these reflect the person making decisions about future care while they are still considered to have capacity and the focus of this review is on current day-to-day care being delivered to the cognitively impaired person. Studies where the shared decisions are made primarily by health or social care staff and, or family carers and do not include the person living with the cognitive impairment will also be excluded. Studies pertaining to participants living with potentially relevant symptoms and/or conditions but without cognitive impairment will be excluded due to the primary focus being on the person living with dementia.

### Types of studies

All empirical study types that meet all other inclusion criteria will be included: randomised controlled trials (RCTs), controlled studies, observational studies and qualitative studies using any recognisable qualitative methodology.

### Outcomes

- Involvement in care planning (eg, as stated within care plans)
- Care delivery congruent with decision made/expressed choice (eg, as stated in daily care records)
- Quality of life for people living with dementia
- Carer satisfaction (staff and/or family carers)
- Well-being for people living with dementia
- Behavioural changes (eg, reduction in behaviours that challenge services)
- Adverse effects (eg, falls, weight loss, adverse outcomes related to medication management)

#### Search strategy

The predefined search strategy is cross-discipline. Limitations have been set with regard to:

- Time—20 years (start date 1996) due to the fast paced nature of treatment and intervention development in this area of care, but to still include the seminal works of Tom Kitwood;
- Language—only studies published in English language will be included;
- Free-text search terms will be limited to title and abstract to promote relevance of search results.

Electronic searches will be performed on the following databases; CINAHL Plus, PubMed, the Cochrane Library, NICE Evidence, OpenGrey, Autism Data, Google Scholar, Scopus and MedicinesComplete. In addition, the reference list of all relevant primary and review articles will be searched manually to identify studies which have not been picked-up by the electronic search. A citation search will also be performed using the ‘cited by’ option on Google Scholar and Scopus, and the ‘related articles’ option in PubMed.

Medical Subject Heading (MeSH) search terms will be combined with Boolean operators AND, and NOT (between columns) to create a search strategy for PuBMED and other electronic databases which recognise MeSH terms. See table 1 columns for MeSH headings and alternative MeSH terms combined with OR for an inclusive search strategy. See table 2 for alternative but equivalent free-text terms operated with ‘wildcards’ and truncations will be used to search CINAHL and other databases which do not recognise MeSH headings.

**Table 1:** Search strategy for databases recognising MeSH headings

Cognition disorders	AND	(Shared decision-making)	NOT	Paediatrics	NOT	Advance directives
Dementia		Decision-making		Children		Advance care planning
Neurocognitive disorders		Patient participation				
Brain injuries		Consumer participation				
Autistic disorder		Cooperative behaviour				
Learning disorders		Decision support				
Stroke						

**Table 2:** Search strategy for CINAHL and databases not recognising MeSH headings using equivalent free-text terms operated with ‘wildcards’ and truncations

Cogniti* Disorder*	AND	Shared decision-making	NOT	Paed*	NOT	Advance directives
Dementia*		Deci* Mak*		Child*		Advance* care planning
Alzheimer*		Patient Participat*				Advance* deci*
Neurocogniti* Dis*		Consumer Participat*				
Brain Injur*		Cooperat*				
Autis*		Decision Support				
Learning Dis*						
Stroke						

*is applied as a truncation mark to the word stem to broaden search terms.

### Study screening and data extraction

Electronic search results will be downloaded into EndNote bibliographic software and duplicates removed where possible. Initially all titles and abstracts retrieved by electronic searches will be screened by one reviewer (RD) against the predefined inclusion criteria and a second reviewer (FB) will independently screen 10% of records to check for consensus. Full-text manuscripts of all potentially relevant citations will be obtained. Hard copies will then be screened independently by RD and either FB or CG. Any disagreements will be resolved by discussion and consensus.

Data will be extracted on the following: (1) the author(s), (2) publication year, (3) country, (4) type of study design, (5) aim(s) and research questions, (6) type of participants and sample size, (7) data collection method (ie, measure of shared decision-making/patient activation/patient involvement), (8) response rate, (9) method(s) of analysis, (10) outcomes.

Additional information will be collected relating to the accessibility and characteristics of interventions, duration of follow-up and any unexpected supplementary findings/outcomes identified by the researcher.

### Quality assessment

Quality assessment will be undertaken by one reviewer (RD), with 10% checked by a second reviewer (FB/CG). RCTs and controlled studies will be assessed using the Cochrane risk of bias tool,^31^ observational studies using the Centre for Evidence Based Management assessment tool and qualitative studies using JBI System for the Unified Management of the Assessment and Review of Information (JBI SUMARI)—Qualitative Assessment and Review Instrument (Qari) framework, which has been identified as one of the most coherent critical appraisal tools to facilitate an assessment of qualitative research validity.^32^

### Analysis

#### Quantitative studies

Results from all studies will be reported in a narrative format. In addition, if there is sufficient homogeneity, and if relevant studies are available, RCTs will be pooled in a meta-analysis with dichotomous outcomes presented as relative risks and continuous data as mean differences, both with 95% CIs. Heterogeneity will be assessed using the χ^2^ test and I^2^ test.^31^ However, in the likely event of heterogeneity (or few RCTs being found) studies will not be pooled, but data will be presented in a narrative format with an indication of whether the effect of the intervention was positive, negative or not statistically significant.

#### Qualitative studies

Review findings will be compiled and evaluated using thematic analysis. This is a widely recognised process,^32–34^ which involves using recurrent themes in primary studies to synthesise new qualitative evidence.^35^ All relevant ‘data’ will be considered for synthesis including those data labelled as quotes, ‘findings’ and ‘results’ as per guidance.^36^

#### Amendments

If any amendments to the protocol are required, they will be individually described, dated and rationalised to ensure transparency and enable the reader to identify potential bias and to replicate the searches if required.

## Discussion

In the UK, there are estimated to be 850 000 people living with dementia and ∼450 000 people living in care homes, the majority of whom have significant memory problems.^4^ An understanding of each person's needs and desires is important to include them in the decisions that shape the decisions about their health and care^5^ ^6^ which is a moral imperative.

Little is known about how decision-making between people living with dementia in extended care settings is shared with their staff and family carers, and further research has been recommended, for example.^16^ ^37^ With a view to adding to the body of knowledge, this review will build on the evidence about the measures, tools and resources from different specialties and aims to bring together all relevant evidence rather than focusing on any specific field of practice, thereby expediting an integrated and interdisciplinary approach to research into dementia care centring around the individual reflecting the comparable drive in health and social care practice (eg, WHO global strategy on people-centred and integrated health services).^26^

The systematic review will provide a contemporary synthesis of evidence in relation to the current understanding of shared decision-making policy and practice for people living with a cognitive impairment in extended care settings. It is designed to explore the characterisation and constructs of shared decision-making for people living with dementia and their carers recognising relationships in care and how those relationships impact on care choices and decisions. Facilitators and barriers and risk and benefits will be explored in the context of resources, methods and tools in an effort to identify a readily available and financially viable intervention that can be independently trialled with a view to comprehensive and equitable implementation throughout the care sector.

It is recognised that there are potential limitations in relation to reporting bias at study and outcome level, for example, unsuccessful implementation studies are less likely to be published by authors; and at review level, for example, the inability to retrieve all relevant research due to possible inadequacies in the search strategy; or reporting bias related to data extraction or analysis. In an effort to overcome these limitations, the search terms and strategy have been reviewed to facilitate a wider breadth of results (eg, databases from a number of professional fields and have been searched to allow for publication bias). The findings will inform the design of an intervention study facilitating shared decision-making for people living with dementia in care homes and have the potential to inform future policy and practice.
